# Revealing the Unseen: Detecting Negative Symptoms in Students

**DOI:** 10.3390/jcm13061709

**Published:** 2024-03-15

**Authors:** Lucie Métivier, Maxime Mauduy, Hélène Beaunieux, Sonia Dollfus

**Affiliations:** 1 Neuropresage Team, Normandie University, UNICAEN, PhIND, UMR-S 1237, GIP Cyceron, Campus Jules Horowitz, 14000 Caen, France; metivier@cyceron.fr; 2 Fédération Hospitalo-Universitaire (FHU A2M2P), Normandie University, UNICAEN, GIP Cyceron, 14000 Caen, France; maxime.mauduy@u-paris.fr (M.M.); helene.beaunieux@unicaen.fr (H.B.); 3 Laboratoire de Psychologie Sociale, Contextes et Régulation (LPS, UR 4471), Université Paris Cité, 92100 Boulogne-Billancourt, France; 4 Laboratoire de Psychologie Caen Normandie (LPCN, EA 7452), Normandie University, UNICAEN, 14000 Caen, France; 5 Service de Psychiatrie, Centre Esquirol, CHU de CAEN Normandie, 14000 Caen, France; 6 Normandie University, UNICAEN, UFR Médecine, Campus 5, 14000 Caen, France

**Keywords:** negative symptoms, ultra-high risk of psychosis, major depressive disorder

## Abstract

**Background**: The transnosographic nature of negative symptoms (NS) makes them fruitful for detecting psychiatric symptoms. The main objective of this study was to determine whether NS can be useful in screening for ultra-high risk of psychosis (UHR) or depressive symptoms in the no-help-seeking student population. The most prevalent NS and their relationship with cannabis use were also researched. **Methods**: From an online survey sent to students, 2128 filled out the Self-evaluation of Negative Symptoms (SNS), Prodromal Questionnaire 16 (PQ-16), Beck Depression Inventory (BDI), and Cannabis Abuse Screening Test (CAST). **Results**: 367 students (17.2%, 95% CI [15.6–18.9]) were considered to have UHR (PQ-16 distress score ≥ 9) and/or depression (BDI ≥ 16). The receiver operation characteristic curve showed that a threshold of 13 for the SNS score discriminated this subgroup of students with a sensitivity and specificity of 79.8% and 70.6%, respectively. The motivational dimension was overrepresented and linked to cannabis use. **Conclusions**: The early detection of NS in the no-help-seeking student population is relevant for detecting depressive and prodromal symptoms. This will enable early intervention to limit the progression to chronic mental disorders. The predominance of NS linked to the amotivational dimension was observed and related to cannabis use.

## 1. Introduction

University students have a higher risk of developing psychiatric illnesses due to the brain’s maturation process and exposure to multiple stressors [1]. Cannabis stands out as the most extensively consumed psychoactive substance, with a 4% world consumption rate and a slightly higher prevalence of 5.4% in Europe [2]. In French universities, one-third of students use cannabis [3], despite its harmful effects on health [4,5]. In addition, the World Mental Health Survey highlighted that 20.3% of students in 21 different countries suffer from at least one mental disorder over a 12-month period [6], and 25% of them have of depression [7], with an increased risk of suicide [8]. In Europe, the prevalence of psychotic symptoms, including ultra-high risk of psychosis (UHR), is 13.8% in young adults (age 16–40 years) [9]. An average of 22% to 38% of individuals with UHR will develop a psychotic disorder within 3 years [10], with an increased risk of transition among cannabis users [11]. The duration of untreated psychosis is associated with a pejorative course of the psychotic disorder [12,13], and detection of UHR maximizes the benefits of early interventions [10] and limits the risk of transition [14]. Negative symptoms (NS) are the first signs to appear in UHR [15,16,17], and their severity is associated with the transition to a first episode of psychosis [10,18,19]. NS are also present in approximately 20% of the general young (age 25–34 years) and adolescent population, at least in an attenuated form [20,21] and in disorders other than schizophrenia [22]. Though primary NS are inherent in schizophrenia, secondary NS in patients with psychotic disorders can be caused by positive symptoms, depression, and cannabis, which can induce an amotivational syndrome [23,24]. Moreover, NS can be described through the motivational and emotional expression dimensions [25], which can also be impaired in depression. As NS overlap with depressive symptoms and appear early in UHR, using a tool that assesses the severity of NS could be helpful in first-line health care.

There are many scales based on observer ratings for the assessment of NS in patients with schizophrenia spectrum disorders [24,26,27,28,29,30], and two are recommended by the European Psychiatric Association: the Brief Negative Syndrome Scale (BNSS) [31] and the Clinical Assessment Interview for Negative Symptoms (CAINS) [32]. The Structured Interview for Prodromal Symptoms (SIPS) [33] and the Comprehensive Assessment of At-Risk Mental States (CAARMS) [34] are used for UHR. However, these last tools were developed primarily for the assessment of attenuated psychotic symptoms and only some aspects of NS are captured [32]. Moreover, all of these scales require a clinical interview with a trained psychiatrist and are not adapted for screening NS. In contrast, self-evaluations involve the person who fills out the form and do not need an interview. In addition, self-assessment is efficient and seems to be more appropriate for detecting symptoms at an early stage. Self-reports have been developed for evaluating psychotic symptoms in schizophrenia [35] and prodromal psychotic symptoms [36,37], but self-assessments for screening NS have not yet been used.

Therefore, we propose using self-evaluation of NS (SNS) to screen no-care-seeking students. The SNS presents good psychometric properties and is very easy to complete [38,39]. In addition, the SNS has demonstrated its validity in screening NS, not only in subjects with schizophrenia [40] or first psychiatric episode [41] but also in the general adolescent population [21]. For all of these reasons, the objectives of this study were to determine whether NS assessed by the SNS can be useful for screening UHR or depressive symptoms in a no-help-seeking student population. We also researched the most prevalent negative symptoms and their relationship with cannabis use since amotivational syndrome can be observed in healthy subjects with chronic cannabis use [23]. 

## 2. Method

### 2.1. Population

This study is part of a larger study exploring substance consumption among students at Caen University (France) (ADUC project) [42]. An online survey, written in French, was created using the Limesurvey application version 6.4.10 (https://limesurvey.org (accessed on 10 November 2020)) [43] and hosted by the university server. It was sent to all students at the University of Caen (*N* = 30,161) via the student mailbox between November and December 2020, during the COVID-19 pandemic, with guaranteed anonymity. One mail reminder was dispatched during this period to encourage student participation.

### 2.2. Ethics

All participants took part in the study voluntarily and gave their consent before starting the survey. The protocol was approved by the Data Protection Officer (DPO) of the university, and the participants’ anonymity was guaranteed by the University Information System Direction (DSI). The study was approved by the French Data Protection Authority on 7 April 2017 (Commission Nationale de l’Informatique et des Libertés- CNIL; n°: u24-20171109-01R1). It was conducted in full agreement with the Declaration of Helsinki (2008) and the ethical standards set by the university’s Psychology Department, which follow the American Psychological Association Ethical Principles of Psychologists and the Code of Conduct for the ethical treatment of human participants [44].

### 2.3. Assessments

The assessments used in the present study collected sociodemographic characteristics (age, gender), cannabis and tobacco use, and self-evaluations of psychiatric symptoms. 

The Cannabis Abuse Screening Test (CAST) [45] was used to assess the presence of a cannabis use disorder by considering the frequency of consumption and five harm-related items: the prevalence of non-recreational use, memory impairment, inability to reduce or stop use, and problems associated with use. The overall score ranges from 0 to 24 (Cronbach’s α = 0.748). If the score is ≥2, it reflects harmful use of cannabis [45]. 

The Cigarette Dependance Scale (CDS) [46] was used to assess nicotine dependence by means of five items: the degree of dependence assessed by the user, number of cigarettes per day, the time of the first cigarette after waking up, assessment of the difficulty to quit smoking, and the number of hours to feel the irresistible urge to smoke. The total score ranges from 5 to 25 (Cronbach’s α = 0.840) [46]. The CDS has shown high internal consistency, good predictive validity, and high test/retest reliability [46,47,48], overcoming the psychometric limitations of the Fagerström Test for Nicotine Dependence. 

The SNS [38] (available on demand via the website https://sns-dollfus.com (accessed on 25 August 2023)) contains 20 short sentences covering the five domains of NS: social withdrawal (items 1 to 4), reduced emotional range (items 5 to 8), alogia (items 9 to 12), avolition (items 13 to 16), and anhedonia (items 17 to 20) (Cronbach’s α = 0.784) [38]. The emotional expression dimension of NS was defined by adding the SNS sub-dimensions “reduced emotional range” and “alogia”, with a score ranging from 0 to 16. Similarly, the motivational dimension of NS was defined by adding the SNS sub-dimensions “social withdrawal”, “avolition”, and “anhedonia”, with a score ranging from 0 to 24. For each sentence of the scale, the participant placed a cross in a box next to the response that best corresponds to their current feelings: 2 (strongly agree), 1 (somewhat agree), or 0 (strongly disagree). The total score is the sum of the 20 scores, ranging from 0 (no NS) to 40 (severe NS) (Cronbach’s α = 0.867) [38]. The SNS has shown high internal consistency, good predictive validity, and high test/retest reliability [38,39,40]. 

Prodromal Questionnaire 16 (PQ-16) [49] contains 16 items: 9 items cover perceptual abnormalities, 5 unusual thought content and paranoia, and 2 concern NS. Each answer is marked true/false, with endorsed symptoms rated on a scale of distress ranging from 0 (no distress) to 3 (severe). The PQ-16 can be scored by a sum of the distress scores (range 0–48), or the total number of symptoms endorsed (range 0–16) (Cronbach’s α = 0.774) [49]. In non-help-seeking settings, a PQ-16 distress score ≥ 9 appears to be more appropriate for distinguishing patients with UHR. Using the distress scale rather than the total symptom score may improve the accuracy of the scale in the non-help-seeking population [50].

The Beck Depression Inventory (BDI) [51] contains 13 questions. Each answer is scored from 0 to 3. The total score ranges from 0 to 39 (Cronbach’s α = 0.880) [52] and reflects the severity of the depression. A score ≤ 4 corresponds to an absence of depression, between 5 to 7 mild depression, and between 8 to 15 moderate depression. A score ≥ 16 corresponds to major depressive disorder [51]. The BDI has shown high internal consistency, good predictive validity, and high test-retest reliability [52].

### 2.4. Planned Statistical Analysis

First, we aimed to detect students with UHR or major depressive disorders using the SNS. Therefore, a receiver operating characteristic (ROC) analysis was carried out to assess the performance of the SNS in discriminating students with potential major depressive disorder and/or UHR. The best threshold was determined by sensitivity and specificity with Youden’s index [53]. The area under the ROC curve evaluated the discriminant performance of the SNS. 

Second, we assessed the frequency of NS among university students using the SNS. NS were considered present when scoring ≥ 2 on any SNS sentence. The frequency of students presenting with NS was calculated and reported for each sentence. 

Third, we aimed to evaluate whether cannabis use, notably beyond depressive and prodromal symptom effects, could explain NS in the student population. Therefore, stepwise linear regressions were performed on SNS total scores, with CAST, CDS, BDI, and PQ-16 distress scores as predictors. Given the potential differential impact of the predictors on the two dimensions of NS, stepwise linear regressions were also performed on SNS emotional expression and motivational dimensions. In addition to controlling for depressive and prodromal symptom effects, we also controlled for the effect of tobacco use (via the CDS scores) to assess the specific effect of cannabis use on NS because tobacco and cannabis use are known to be strongly intertwined [54]. To correct for the non-normality of our variables due to their positive skewness, we took their square root [55]. 

All *p*-values were considered significant if <0.05. All statistical analyses were conducting using Jamovi 2.2.5 software.

## 3. Results

Of the 30,161 students who received the online survey, participants who filled out the SNS, PQ-16, BDI, and CAST were included in the present study. After the exclusion of 75 outliers, determined by applying the interquartile range method to the total SNS scores [56], 2128 students were included in the analyses. The sociodemographic and clinical characteristics, as well as cannabis and tobacco consumption, are described in Table 1.

### 3.1. ROC Analysis of Total SNS Score

A total of 269 (12.6%; 95% CI [11.2–14.1]) students had a PQ-16 distress score ≥ 9 and may be considered as UHR, 194 (9.1%. 95% CI [7.9–10.3]) students had a BDI score ≥ 16 and may present with major depressive disorder, and 367 (17.2%; 95% CI [15.6–18.9]) students had one or both conditions. 

**Figure 1:**
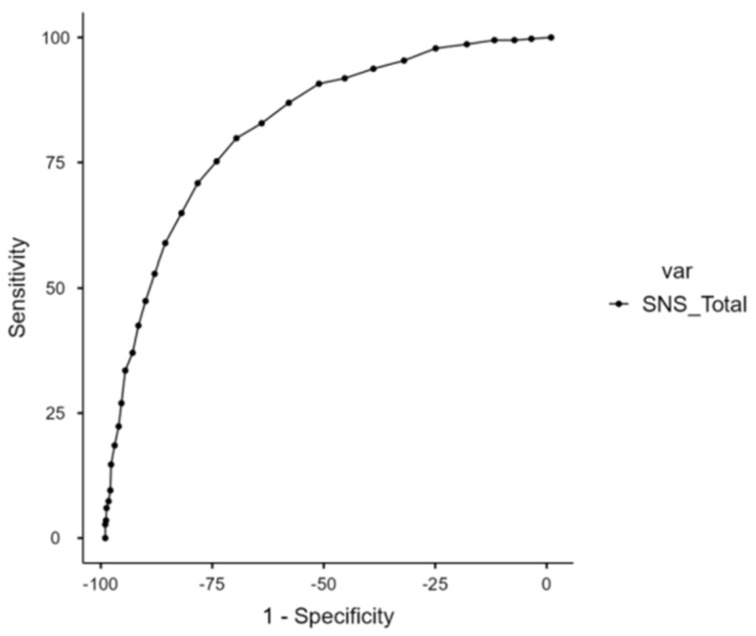
Receiver operating characteristic curve of the screening performance of SNS in discriminating participants with BDI score ≥ 16 and/or PQ-16 distress score ≥ 9. SNS: Self-Evaluation of Negative Symptoms; var: variation.

ROC analysis was performed to assess the performance of the SNS in screening subjects with potential UHR and/or major depressive disorder. The ROC curve (Figure 1) showed a significant area of 0.82, with a cutoff point at 13 (Youden’s index = 0.504) and with sensitivity and specificity of 79.84% and 70.58%, respectively. Various thresholds according to the sensitivity and specificity are provided in Table 2.

### 3.2. Frequency of Negative Symptoms in the Student Population 

The frequency of NS across the two dimensions is provided in Table 3. Students having NS related to the motivational dimension with a predominance of amotivational symptoms (items 14 and 15; 24.4% (95% CI [22.6–26.3]) and 29.6% (95% CI [27.6–31.5]), respectively) are overrepresented.

### 3.3. Stepwise Linear Regressions 

#### 3.3.1. Association of the Total SNS Score with Cannabis Use, Tobacco Use, and Clinical Variables

The stepwise linear regression retained a three-variable model, including BDI, PQ-16 distress, and CAST. The CDS did not significantly improve the prediction of NS beyond the three-variable mode. Globally, the three-variable model was significant and explained 36.6% of the variance in total SNS scores (*R*^2^ = 0.366, *F*(3,98) = 18.9, *p* < 0.001). More precisely, BDI (std.β = 0.42, SE = 0.07, 95% CI = [0.25, 0.60], *r*^2^ = 0.277, *p* < 0.001), PQ-16 distress (std.β = 0.22, SE = 0.19, *95%* CI = [0.04, 0.40], *r*^2^ = 0.047, *p* = 0.017), and CAST (std.β = 0.21, SE = 0.18, 95% CI = [0.05, 0.37], *r*^2^ = 0.042, *p* = 0.012) were positively associated with NS (Table 4).

Thus, higher levels of depressive symptoms, prodromal symptoms, and cannabis use increased the severity of NS in the student population.

#### 3.3.2. Association of the SNS Motivational Dimension with Cannabis Use, Tobacco Use, and Clinical Variables

The stepwise linear regression retained a three-variable model, including BDI, PQ-16 distress, and CAST. The CDS did not significantly improve prediction of the motivational dimension of NS beyond the three-variable mode. Globally, the three-variable model was significant and explained 51.2% of the variance in the SNS motivational dimension (*R^2^* = 0.512, *F*(3,98) = 34.3, *p* < 0.001). More precisely, BDI (std.β = 0.45, SE = 0.05, *95%* CI = [0.29, 0.60], *r^2^* = 0.363, *p* < 0.001), PQ-16 distress (std.β = 0.35, SE = 0.15, *95%* CI = [0.19, 0.50], *r*^2^ = 0.110, *p* < 0.001), and CAST (std.β = 0.20, SE = 0.14, *95%* CI = [0.06, 0.34], *r*^2^ = 0.039, *p* = 0.006) were positively associated with the motivational dimension of NS (Table 4). 

Thus, higher levels of depressive symptoms, prodromal symptoms, and cannabis use increased the severity of the motivational dimension of NS in the student population. 

#### 3.3.3. Association of the SNS Emotional Expression Dimension with Cannabis Use, Tobacco Use, and Clinical Variables

The stepwise linear regression retained a one-variable model including BDI. PQ-16 distress, CAST, and CDS did not significantly improve the prediction of the emotional expression dimension of NS beyond the one-variable model. This one-variable model was significant and explained 5.86% of the variance in the SNS emotional expression dimension (*R^2^* = 0.058, *F*(1,100) = 6.22, *p* < 0.001), with BDI scores positively and significantly (std.β = 0.24, SE = 0.07, *95%* CI = [0.05, 0.44], *r^2^* = 0.058, *p* = 0.014) associated with the emotional expression dimension of NS (Table 4). 

Thus, the results indicate that higher levels of depressive symptoms increase the severity of the emotional expression dimension of NS in the student population.

**Table 4:** Stepwise linear regression analysis.

Dependent Variables	Independent Variables	β	Std.β	SE	95% CI	R^2^	*p*
**SNS total score**	BDI total score	0.31	0.42	0.07	[0.25–0.60]	0.277	<0.001
	PQ-16 distress score	0.47	0.22	0.19	[0.04–0.40]	0.047	0.017
	CAST score	0.46	0.21	0.18	[0.05–0.37]	0.042	0.012
SNS motivational dimension	BDI total score	0.30	0.45	0.05	[0.29–0.60]	0.363	<0.001
	PQ-16 distress score	0.68	0.35	0.15	[0.19–0.50]	0.110	<0.001
	CAST score	0.41	0.20	0.14	[0.06–0.34]	0.039	0.006
SNS emotional expression dimension	BDI total score	0.16	0.242	0.07	[0.05–0.44]	0.058	0.014

β: Crude Beta coefficient; BDI: Beck Depression Inventory; CAST: Cannabis Abuse Screening Test; 95% CI: 95% Confidence Interval; *p*: *p*-value; PQ-16 distress score: Prodromal Questionnaire 16 distress score; R^2^: Coefficient of Determination; SE: Standard Error; SNS: Self-evaluation of Negative Symptoms; Std.β: Standardized Beta coefficient.

## 4. Discussion

The present study highlights that NS assessed by the SNS can discriminate students with potential major depressive syndrome and/or those at risk of psychosis and that the frequency of NS in this population remains high, with a predominance of the amotivational dimension, which is related to cannabis use. The following aspects are worth discussing: the interest in the SNS for screening students suffering from potential major depressive disorder and/or UHR; the prevalence of NS, depressive symptoms, and UHR in the student population; and the role of cannabis consumption in understanding NS. 

First, our study’s results show that the SNS has good discriminant properties for screening students with potential major depressive disorder and/or UHR. Above a cut-off point of 13, the SNS discriminated these participants with a sensitivity of 79.84% and specificity of 70.58%. The SNS threshold value was the same as that found in the non-help-seeking adolescent population for screening UHR [21], but, as expected, was greater than that observed in first-episode psychosis (threshold of 11) [41] and chronic schizophrenia populations (threshold of 7) [40]. The intensity of NS is higher and often pathological in people suffering from a first episode of psychosis or schizophrenia than in the general student population, which explains the lower thresholds in these groups. Compared to other tools developed primarily for the assessment of attenuated psychotic symptoms, such as CAARMS or SIPS, the SNS can capture NS. In addition, compared to other evaluations based on observer ratings, the SNS can provide clinical information not necessarily detected by professionals during an interview and can deliver information on the person’s own experience [40,57]. Finally, the SNS is easy to use and does not require external intervention, enabling large-scale detection of NS, depressive symptoms, and UHR. 

The high level of NS found in this study is in agreement with previous studies reporting the presence of NS in the general population [20,21]. Werbeloff et al. (2015) showed that approximately 20% of subjects aged 24 to 34 years had at least one NS assessed with the SANS scale [20]. Rodriguez Testal et al. (2019) observed that 17% of adolescents had a high score (85th percentile) on the SNS [21]. In the present study, the most frequent NS reported by the no-help-seeking student population were related to the motivational dimension, especially avolition (items 14 and 15), which is in line with Rodriguez Testal et al. [21] Both items refer to difficulty being regular in daily activities (item 14) and the lack of motivation to do something (item 15). This difficulty in drawing up an action plan may interfere with the student’s commitment to higher education and academic achievement [58]. The less frequent NS reported by the student population were related to anhedonia (items 17 and 20) and social withdrawal (items 2 and 3). As these last dimensions are preserved in most students, the probability of psychotic disorders in this population is low, despite the strong association between anhedonia and psychosis [59]. 

This study also highlights that 9.1% of students had a potential major depressive disorder, 12.6% potentially had UHR, and 17.6% had one or both disorders. Regarding depression, its prevalence varies between studies. An international study revealed that 4.5% to 7.7% of 14,000 students had symptoms of major depressive disorder [6], whereas a recent meta-analysis reported that 25% of students experience symptoms of depression [7]. The prevalence is even higher in medical students, with 30.2% presenting with depressive symptoms [60], which worsened in university students during the COVID-19 pandemic [61]. In addition to the social and professional consequences, the main risk of depression is suicide and suicidal behavior, with almost 800,000 suicides every year in the world general population [8]. In view of these results, detecting depression in students is a major challenge. Furthermore, in line with our results, the point-prevalence of psychotic symptoms in the young adult community (age 16–40 years) measured by the SIPS has been reported to be 13.8% [9]. An average of 22% to 38% of patients with UHR are estimated to develop a psychotic disorder within 3 years [10]. Consequently, early management of psychoses is a key prognostic factor. 

Here, the severity of NS was associated with the levels of depression, prodromal signs of psychosis, and cannabis use. These results support the transnosographic dimension of NS [22]. In the linear regression model, the load of cannabis use was lower than that of depression and prodromal signs. Cannabis use was also partly responsible for the severity of the motivational dimension, but not the emotional expression dimension. This is in agreement with a meta-analysis showing that cannabis worsened NS in the general population, but with a moderate grade of proof [62]. However, the impact of cannabis use on amotivational syndrome is still controversial. Another study pointed out that decreased motivation among cannabis users has not been clearly established [63]. In a population suffering from schizophrenia, a recent meta-analysis highlighted the absence of a specific association between current cannabis use and the severity of NS [64]. These discrepancies could be explained by different types of populations, which may present different levels of dopaminergic dysregulation [65].

Despite the interesting results discussed above, our study had some limitations. First, the anonymity of the online survey prevented us from confirming the diagnosis of UHR and major depressive disorder established by self-assessments, the PQ-16 and BDI, respectively. However, the thresholds used in this study to define these disorders are those reported in the literature [49,50,51]. Second, the use of SNS was not associated with an assessment based on observer rating. Nevertheless, the SNS is validated, and the scores correlate with the SANS, BNSS, or PANSS negative subscale [38,39]. Third, the cross-sectional nature of this study did not allow us to determine whether the NS are predictive of the transition from UHR to psychosis. Fourth, this work focuses on a student population from a single French university and the generalization and confirmation of the pathological threshold of 13 for the SNS requires replication in populations from different universities and countries. 

## 5. Conclusions

The early detection of pathological NS in the no-help-seeking student population is relevant for the detection of UHR and depressive symptoms, due to the overlap with various diagnostic categories and early onset during psychotic disorders. This detection may enable early medical, psychological, and social intervention and limit the risk of progression to chronic mental disorders. The frequency of NS in this population remains high, with a predominance of the amotivational dimension, which is related to cannabis use.

## Figures and Tables

**Table 1:** Characteristics of the study population.

Variable	*N* = 2128
**Sociodemographic**	
Age, years	19.80 (2.25); [18–34]; (95% CI [19.70–19.90])
Gender, % men	29.1%; (95% CI [27.2–31.0])
**Clinical**	
SNS emotional expression score	4.25 (3.27); [0–16]; (95% CI [4.11–4.39])
SNS motivational score	6.07 (4.47); [0–24]; (95% CI [5.88–6.26])
SNS total score	10.33 (6.77); [0–30]; (95% CI [10.00–10.60])
PQ-16 distress score	3.69 (5.10); [0–46]; (95% CI [3.47–3.90])
BDI	6.74 (6.05); [0–39]; (95% CI [6.48–7.00])
**Toxic consumption**	
CAST scores	0.58 (2.32); [0–24]; (95% CI [0.48–0.68])
CDS	11.9 (4.75); [5–24]; (95% CI [11.4–12.4])

Values are given as mean (SD); [minimum–maximum] unless otherwise noted. BDI: Beck Depression Inventory; 95% CI: 95% Confidence Interval; CAST: Cannabis Abuse Screening Test; CDS: Cigarette Dependance Scale; PQ-16: Prodromal Questionnaire 16; SD: standard deviation; SNS: Self-evaluation of Negative Symptoms.

**Table 2:** Receiver operating characteristic analysis discriminating participants with negative symptoms and BDI score ≥ 16 and/or PQ-16 distress score ≥ 9.

SNS Cut-Off	Sensibility (%)	Specificity (%)	Youden’s Index	AUC
**10**	90.74	52.07	0.428	0.823
**11**	86.92	58.83	0.458	0.823
**12**	82.83	64.91	0.477	0.823
**13**	**79.84**	**70.58**	**0.504**	**0.823**
**14**	75.20	75.01	0.502	0.823
**15**	70.84	79.27	0.501	0.823
**16**	64.85	82.91	0.478	0.823
**17**	58.86	86.54	0.454	0.823

AUC: area under the curve; BDI: Beck Depression Inventory; SNS: self-evaluation of negative symptoms.

**Table 3:** Frequency of students with a score of 2 on each SNS item (*N* = 2 128).

NS Dimension	SNS Subscores	SNS Item	Mean (SD); [Minimum–Maximum]	Percentage of Affirmative Responses (Score = 2)
**Motivational** **dimension**	**Social withdrawal**	**1**	0.786 (0.682); [0–2]	14.8%, (95% CI [13.3–16.3])
		**2**	0.319 (0.578); [0–2]	5.9%, (95% CI [4.7–6.8])
		**3**	0.221 (0.491); [0–2]	3.4%, (95% CI [2.7–4.2])
		**4**	0.541 (0.696); [0–2]	11.8%, (95% CI [10.4–13.1])
	**Avolition**	**13**	0.721 (0.696); [0–2]	14.2%, (95% CI [12.7–15.6])
		**14**	0.953 (0.730); [0–2]	24.4%, (95% CI [22.6–26.3])
		**15**	1.011 (0.762); [0–2]	29.6%, (95% CI [27.6–31.5])
		**16**	0.540 (0.708); [0–2]	12.6%, (95% CI [11.2–14.1])
	**Anhedonia**	**17**	0.236 (0.493); [0–2]	3.1%, (95% CI [2.4–3.9])
		**18**	0.248 (0.513); [0–2]	3.8%, (95% CI [3.0–4.64])
		**19**	0.246 (0.499); [0–2]	3.2%, (95% CI [2.4–3.9])
		**20**	0.279 (0.564); [0–2]	5.8%, (95% CI [4.8–6.8])
**Emotional** **expression** **dimension**	**Reduced emotional range**	**5**	0.539 (0.703); [0–2]	12.3%, (95% CI [10.9–13.7])
		**6**	0.428 (0.630); [0–2]	7.6%, (95% CI [6.4–8.7])
		**7**	0.296 (0.581); [0–2]	6.5%, (95% CI [5.4–7.5])
		**8**	0.824 (0.761); [0–2]	21.7%, (95% CI [19.9–23.4])
	**Alogia**	**9**	0.712 (0.777); [0–2]	19.9%, (95% CI [18.2–21.6])
		**10**	0.427 (0.655); [0–2]	9.2%, (95% CI [7.9–10.4])
		**11**	0.555 (0.734); [0–2]	14.6%, (95% CI [13.1–16.1])
		**12**	0.492 (0.693); [0–2]	11.5%, (95% CI [10.1–12.9])

Values are given as mean (SD); [minimum–maximum]. 95% CI: 95% Confidence Interval; NS: negative symptoms; SD: standard deviation; SNS: self-evaluation of negative symptoms.

## Data Availability

The data that support the findings of this study are available on request from the corresponding author. The data are not publicly available due to privacy or ethical restrictions.
